# Facile strain analysis of largely bending films by a surface-labelled grating method

**DOI:** 10.1038/srep05377

**Published:** 2014-06-20

**Authors:** Norihisa Akamatsu, Wataru Tashiro, Keisuke Saito, Jun-ichi Mamiya, Motoi Kinoshita, Tomiki Ikeda, Jun Takeya, Shigenori Fujikawa, Arri Priimagi, Atsushi Shishido

**Affiliations:** 1 Chemical Resources Laboratory, Tokyo Institute of Technology, R1-12, 4259 Nagatsuta, Midori-ku, Yokohama 226-8503, Japan; 2 Department of Advanced Materials Science, Graduate School of Frontier Sciences, The University of Tokyo, 5-1-5 Kashiwanoha, Kashiwa-shi, Chiba 277-8561, Japan; 3 International Institute for Carbon-Neutral Energy Research (WPI-I2CNER), Kyushu University, 744 Motooka, Nishi-ku, Fukuoka 819-0395, Japan; 4 Department of Applied Physics, Aalto University, P.O. Box 13500, FI-00076 Aalto, Finland; 5 Current address: Saitama Institute of Technology, 1690 Fusaiji, Fukaya, Saitama 369-0293, Japan.; 6 Current address: Chuo University, 1-13-27 Kasuga, Bunkyo-ku, Tokyo 112-8551, Japan.

## Abstract

Mechanical properties of flexible films, for example surface strain of largely bending films, are key to design of stretchable electronic devices, wearable biointegrated devices, and soft microactuators/robots. However, existing methods are mainly based on strain-gauge measurements that require miniaturized array sensors, lead wires, and complicated calibrations. Here we introduce a facile method, based on *surface-labelled gratings*, for two-dimensional evaluation of surface strains in largely bending films. With this technique, we demonstrate that soft-matter mechanics can be distinct from the mechanics of hard materials. In particular, liquid-crystalline elastomers may undergo unconventional bending in three dimensions, in which both the inner and outer surfaces of the bending film are compressed. We also show that this method can be applied to amorphous elastomeric films, which highlights the general importance of this new mechanical evaluation tool in designing soft-matter-based electronic/photonic as well as biointegrated materials.

With increasing pressure towards product liability and energy efficiency, advanced materials must meet the requirements of lightness and strength, which in turn requires thorough testing. This highlights the importance of experimental stress analysis and facile techniques for measuring strain at material surfaces. Among various methods to measure strain or deformation based on mechanical, optical, and electrical phenomena^1^, one of the most common methods is the strain-gauge measurement based on strain-dependent electrical resistance in the body to which the device is attached. Nonmetallic semiconductor or metallic foil is bonded to the target with glue, which means that the strain gauge composed of semiconductor or metal and glue is basically suited to strain measurement of hard materials.

On the other hand, recent advances in material and device engineering have resulted in soft, flexible and stretchable devices, enabling wearable, human-body friendly devices^2^,^3^,^4^,^5^,^6^. In such devices, design and integration of appropriate materials with flexibility and softness is essential. This places new importance of strain measurement on largely bending materials; however, soft-matter mechanics have not been properly developed yet, compared to conventional mechanics that deals with hard materials. Elastomers can stretch up to hundreds of percent without fatigue; however, the fracturing of semiconductor layers and metal electrodes already occurs at *ca.* 1% strain^7^,^8^. Strains in soft-matter-based devices are frequently modelled by assuming that they bend in the same way as hard materials, such that their inner surface is compressed while the outer surface is tensiled, with a neutral surface in the centre of the film. But can surface deformations in soft-matter-based films with large bending be evaluated by assuming such conventional deformation characteristics?

In some cases, strain of soft materials can be visualized by other approaches. For instance, external stress can affect the self-assembly or aggregation state of an appropriately designed material system and thus trigger changes in its photophysical, photochemical, or chemoluminescent properties^9^,^10^,^11^,^12^. Such changes can give rise to so-called mechanoluminescence, allowing strain sensing based on colour change^13^. Alternatively, strain-induced colour change can occur in colloidal photonic crystals that are infiltrated with elastomers^14^,^15^. Although such direct-visualization sensing schemes are very attractive, they are usually based on highly elaborate materials designs and require large strains or shear forces. Recently, stretchable electronic systems have been developed by device engineering approach^16^. Bao et al. demonstrated that optically transparent, stretchable films of carbon nanotubes have the ability to detect both pressure and strain^17^. Rogers et al. created 3D elastic membranes shaped precisely to match the epicardium of the heart as a platform for deformable arrays of multifunctional sensors^18^. Strain sensors based on piezoresistive effects in nanomembranes of Si allow monitoring of the mechanics of contractions of the heart. In addition to strain, temperature and pH sensors are integrated. Most recently, multifunctional wearable devices with sensors, memory, and therapeutic modules have been fabricated^19^. Muscle activity is monitored with singe crystal Si nanomembrane strain sensors. Those devices are sophisticated, while the strain measurement relies on semiconductor strain gauge, which still shares the concerns such as the complicated structures including metallic wires and sensor array.

There are optical methods to remotely measure strain based on photoelasticity, holography, Moiré, and so on^1^,^20^,^21^,^22^. They give precise strain measurement, whereas most methods cannot be applied to largely bending films. X-ray diffraction has been utilized to measure strain of bending metal plate by synchrotron radiation^23^,^24^. All in all, a facile method to measure surface strain of largely bending films is desired.

Here we propose a surface-labelled grating method that can be applied to practically any largely bending film with a flat surface, which allows spatially resolved, separate probing of the front and back surfaces in two dimensions. The basic concept relies on a 1D or 2D diffraction grating that is linked selectively to either surface of the target material. The surface strains are then visualized by monitoring the diffraction angles of a probe laser beam. Depending on the material of interest, the grating acting as the surface label can either be inscribed into the surface layer of the material, or alternatively, a separate elastomeric label can be attached onto either of its surfaces. As a model compound for the former, we use photochromic, azobenzene-containing liquid-crystalline elastomers. In the latter case, surface-relief gratings on low-modulus, amorphous poly(dimethylsiloxane) (PDMS) elastomers, in addition to being commonly used as stretchable-electronics substrates^25^,^26^, are suitable as externally attachable surface labels.

## Results and Discussion

**Figure 1:**
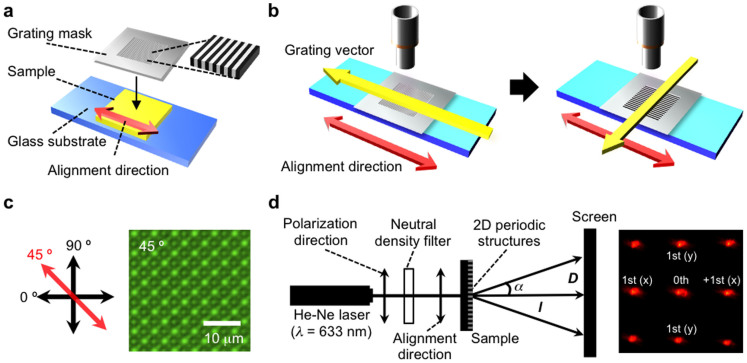
Surface labelling of azobenzene-containing liquid-crystalline elastomer films. (a), A homogeneously aligned azobenzene-containing liquid-crystalline elastomer film is irradiated through a grating mask, causing the photoisomerization-induced periodic change in molecular alignment to show up as a phase grating, a surface-labelled grating (due to the high absorbance of the azobenzene moieties, the grating only forms on the surface layer of the film). (b), Two subsequent exposures with mutually perpendicular grating mask orientations give rise to a two-dimensional surface-labelled grating. (c), A polarized optical micrograph of a two-dimensional surface-labelled grating. (d), The surface-labelled gratings can be used as a surface-specific strain evaluation tool for the films upon mechanical or photochemical bending, by monitoring the changes in the diffraction angle (α) of a probe laser beam. *D* and *l* represent distance between transmitted and first diffracted beams on the screen, and distance between the film and the screen, respectively.

Two reasons render azobenzene-containing liquid-crystalline elastomers as materials of choice for our method's proof of concept. First, the surface labelling is easily achievable by (i) aligning the azobenzene moieties by elongation of the elastomeric film, and (ii) irradiating the aligned sample with UV radiation through a photomask to induce *trans–cis* photoisomerization of azobenzene, which decreases the molecular alignment order within the irradiated areas (Fig. 1a and Supplementary Figs. S1–S3 online). The diffraction grating can be tuned by mechanical stretching^27^,^28^, hence it acts as a simple and remote strain visualization tool by monitoring changes in the diffraction angles of a probe beam transmitted through the grating. We used thick (300 μm) films in the present case. The isomerization-induced diffraction grating was confined to a surface layer of 25 μm due to the high absorbance of azobenzene molecules, as verified by polarized optical microscopy (see Supplementary Fig. S4 online). This confinement allows the strains at the two film surfaces to be studied separately. Importantly, one can also inscribe several gratings on the same area of the film through multiple exposures (Fig. 1b). A 2D grating (allowing a 2D surface-strain analysis) obtained by two exposures with perpendicular photomask orientations is shown in Fig. 1c, and Fig. 1d shows the resultant diffraction pattern of a transmitted He–Ne laser beam formed on a screen. Further details on the materials used, the preparation and properties of the surface-labelled films, and the details of the optical setup are provided in Methods.

Our second motivation for using azobenzene-containing liquid-crystalline elastomers as model compounds is that they possess the unique capability of converting input light energy into mechanical motion^29^. This intriguing characteristic enables the harnessing of light to fuel soft actuators, micromechanical devices, and artificial muscles. Since the initial investigations of photoinduced contraction^30^ and bending^31^ of azobenzene-containing liquid-crystalline elastomers, different types of photoinduced three-dimensional motions, such as high-frequency oscillation^32^, swimming^33^, and robotic arm movements^34^, have been demonstrated. Similar to the formation of the diffraction gratings we use for surface labelling, these motions are triggered by the photoisomerization of azobenzene molecules, which changes the alignment order of the surrounding mesogens and creates macroscopic strains that give rise to macroscopic deformation. Coupling between the optical and mechanical properties and the resultant dynamics of the macroscopic deformation can be highly complex in such systems, as shown both theoretically^35^,^36^ and experimentally^37^,^38^. However, to the best of our knowledge, the actual surface-specific strains caused by photoinduced deformation have not been evaluated to date. The surface-labelled grating method is a perfect tool for this, as it may allow further insight into the details of light-triggered macroscopic motions and actuation.

**Figure 2:**
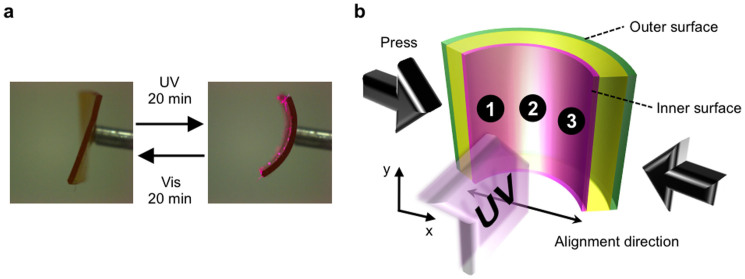
Photoinduced bending of azobenzene-containing liquid-crystalline elastomer films. (a), Upon irradiation with UV light, a homogeneously aligned azobenzene-containing liquid-crystalline elastomer film bends towards the light source along the molecular alignment direction. The initial flat state can be restored by irradiating the film with visible light. (b), The surface-labelled grating allows for positional mapping of the surface strains at both the inner and outer surfaces of the bending film.

We used surface-labelled gratings to compare the surface strains from the two-dimensional bending of azobenzene-containing liquid-crystalline elastomer films under mechanical stress and UV irradiation. For the latter, the (asymmetric) strain originates from a gradient in the photoisomerization-induced decrease in the molecular alignment order; the resultant bending as well as the subsequent visible-light-induced restoration of the initial flat state is illustrated in Fig. 2a.

Photochemically induced molecular alignment changes do not occur in the case of external mechanical stimulus, and an evaluation of the surface strains between these two cases may show that there are significant differences in their surface deformations. The strain analysis principle is schematically illustrated in Fig. 2b. In addition to surface specificity and the possibility of two-dimensional evaluation, the method allows us to spatially resolve the strain distribution with high accuracy. The whole set of mechanically and photochemically induced strains for the three distinct measurement points marked in Fig. 2b is provided in Supplementary Fig. S5 online; herein, we concentrate on the most distinctive features between these two stimuli and we focus our consideration on point 2.

**Figure 3:**
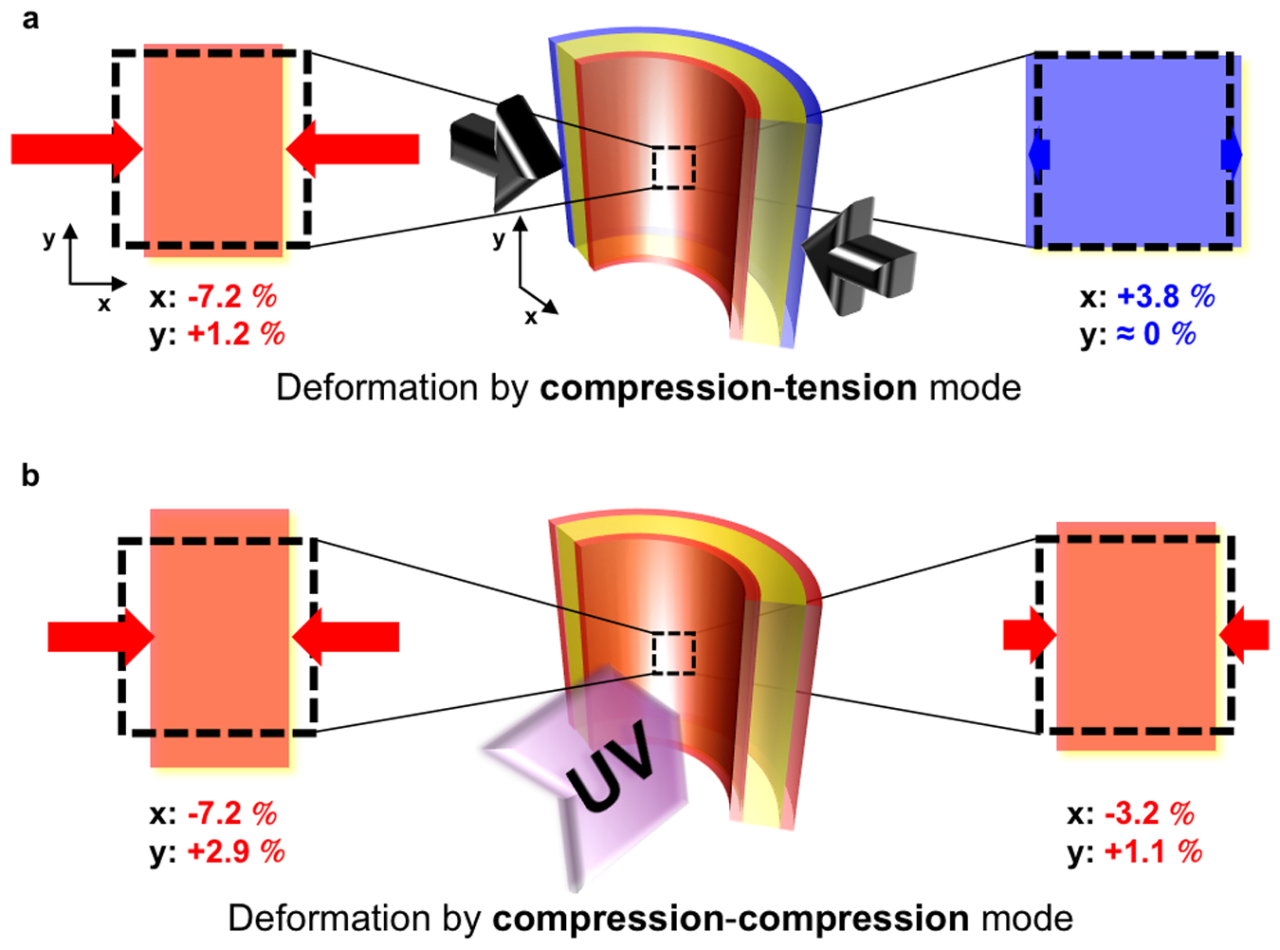
Surface-strain analysis of mechanically and photochemically bent azobenzene-containing liquid-crystalline elastomer films at the same radius of curvature. (a), Upon mechanical bending, the inner surface of the film contracts and the outer surface expands along the bending direction. (b), Upon photoinduced bending, both the inner and outer surfaces contract, which highlights that the bending of soft matter can be distinct from that of hard matter.

The essence of the surface-strain evaluation for the azobenzene-containing liquid-crystalline elastomer film is summarized in Fig. 3. Under external mechanical stress, the surface strains that emerge can be considered “conventional”, in that the inner surface of the film is compressed in the *x*-direction (in this particular case by 7.2%). This surface correspondingly becomes tensile in the *y*-direction by 1.2% to minimize the volume change. At the same time, the outer surface expands by 3.8% in the *x*-direction, whereas no change is observed in the perpendicular direction. Upon light stimulus, the mechanical response of photoresponsive LCEs became highly complex due to changes in the molecular conformation and alignment order, which in turn may change, *e.g.*, the free volume of the material. Indeed, unlike compression–tension mode bending observed under mechanical stress, light-induced bending results in the compression of both the inner and outer surfaces in the direction of molecular alignment (*x*-direction), by 7.2% and 3.2%, respectively. The corresponding expansions in the perpendicular direction are 2.9% and 1.1% for the inner and outer surfaces, respectively. The compression–compression mode bending occurs even though the penetration depth of the incident UV light is less than 10% of the film thickness. This highlights the unique coupling between the optical and mechanical properties of photochromic liquid-crystalline elastomers through cooperative motions.

When the azobenzene-containing liquid-crystalline elastomer film is irradiated with UV light, the film bends toward the light source due to surface contraction. According to the mechanics of conventional hard materials, this should result in the expansion of the outer surface. In fact, the assumption that a neutral surface with zero strain is maintained in the centre of the film has been applied to the bending of flexible polymer films^39^. However, as observed here, the outer surface might even contract as a consequence of inner surface compression. Even if the visual appearances of the mechanically and photochemically bent films are identical, their surface strains, detected by the surface-labelled gratings, are very distinct since the bending mechanisms are different. Moreover, even for mechanically bent film the neutral surface is shifted towards the outer surface; this suggests that assumptions made based on hard matter are not eligible in the case of soft matter. The surface-labelled grating method provides a facile evaluation tool for a more detailed understanding of such issues.

**Figure 4:**
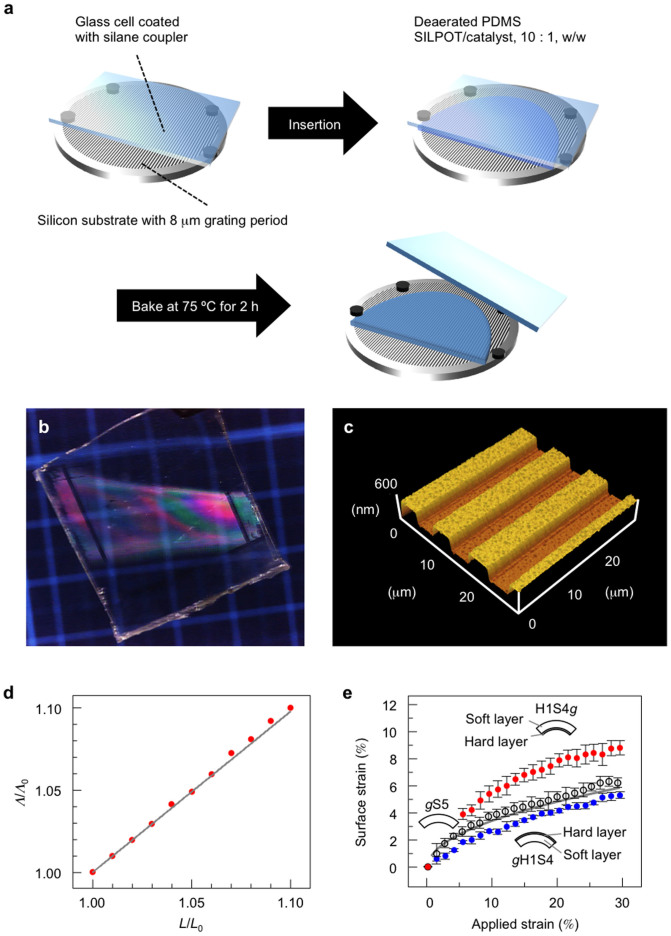
Surface labelling based on surface-relief gratings in PDMS films. (a), Fabrication principle of PDMS-based surface-labelled gratings. For single-layer films, the glass cell is prepared using 500 μm spacers between a glass substrate and a silicon substrate, for a surface-relief grating with 8 μm periodicity. A mixture of Sylpot and catalyst (10:1 (w/w) for the soft film and 5:1 (w/w) for the hard film) was injected into the cell by capillary force and baked for 2 h at 75°C. After removing the film from the substrates, a free-standing PDMS film was obtained. The detailed procedure is shown in Supplementary Fig. S6 online. (b), Photograph of a 500-μm thick surface-labelled PDMS film. (c), An AFM image of a surface-relief grating on a PDMS film. (d), Simultaneous measurement of the strains generated under external mechanical stress using a thermomechanical analyzer (*L*/*L*_0_). The surface-labelled grating method (*L*/*L*_0_) shows that the strains match perfectly at least up to 10.0%. (e), Strains at the outer tensile surfaces of mechanically bent single-layer and bilayer (with hard and soft layers) PDMS films as a function of the ratio of pressed distance to the initial film length (applied strain). The grey line is the calculated surface strain by equation (1)^9^. Mean values of n = 5 and 3 films are plotted for single-layer and bilayer films, respectively. Error bars represent standard deviation.

Although convenient, surface-labelled gratings based on periodic molecular alignment change can only be inscribed in photochromic materials, which is a limiting factor. Hence, to generalize the method we use PDMS-based relief gratings as surface labels, since they can easily be attached to (and removed from) virtually any surface in order to perform a strain analysis (*e.g.*, in stretchable electronics). The PDMS gratings can be conveniently fabricated by adapting techniques from a soft-lithographic approach^40^ (Fig. 4a), *i.e.*, by (i) adding a layer of prepolymer over a silicon master relief (the layer thickness can be controlled with appropriate spacers and a cover glass); (ii) polymerizing at elevated temperature to obtain the solid, crosslinked PDMS elastomer; and (iii) releasing the elastomeric surface-relief grating from the master. Such PDMS labels are isotropic, transparent, and of high optical quality (Fig. 4b). A 3D atomic force microscopy (AFM) image of an example PDMS grating is shown in Fig. 4c. At this stage we have only used a 1D surface label to validate the concept; however, it is straightforward to generalize the analysis to 2D by simply using a different master relief. An additional benefit for strain analysis is the softness of PDMS (elastic modulus *ca.* 2.1 MPa), which ensures that the surface label does not disturb the strain evaluation of the target material.

Figure 4d shows control measurements in which the strain (under external mechanical stress) is simultaneously monitored using a thermomechanical analyzer (TMA) and the surface-labelled grating method. The strain values measured with these two independent methods match perfectly up to at least 10.0% strain, which is the limit of our TMA instrument. Figure 4e provides the strains at the outer tensile surfaces of mechanically bent soft single-layer (elastic modulus: 0.44 MPa) PDMS films, as well as a bilayer PDMS film composed of hard and soft layers. All the bent films showed the same shape (see Supplementary Fig. S7 online).

For the single-layer PDMS films (gS5), the surface strains can be considered “conventional”, *i.e.*, the surface strains measured by the surface-labelled grating almost agree with those calculated by equation (1) employed by Rogers and coworkers^7^: 
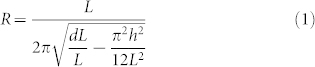
where *R* is the radius curvature, *L* is the film length, *dL* is the compressed distance, and *h*_s_ is the film thickness.

In contrast, the bilayer films exhibited anomalous tensions compared to the single-layer PDMS film. The hard layer in the surface decreases the surface strain in outward bending (gH1S4); however, the surface strain is increased in inward bending of the hard layer (H1S4g). Hence, the surface strains depend markedly on the details of the materials design, as can be conveniently monitored with the surface-labelled grating method. The bent shape of bilayer films was the same in appearance as mentioned above. Hence the equation that describes the bent shape of single-layer films with the sinusoidal curve might be also applied to the bilayer films. It is noteworthy that, unlike with conventional mechanics calculation, this method of analyzing the surface strain does not require knowledge of any materials properties, such as the film thickness and Young's modulus. This provides us with a facile analysis technique for multiple-layered films composed of various organic and inorganic materials. We believe this method is a useful tool for use in surface-strain control for flexible films. For example, it allows us to design surface-strain free bending films or films with positionally controlled surface strain, which are important for flexible electronics and biomedical applications. Finally, a strain analysis of a mechanically-bent poly(ethylene naphthalate) (PEN) film labelled with a PDMS layer is shown in Supplementary Fig. S8 online. This result also serves to highlight the method's ability for analyzing the surface strains of various films that may consist of metal, glass, and hybrid materials, in addition to common polymer films. As long as the film surface is flat enough to transmit or reflect a probe beam, strain can be measured even with light absorbing materials. Resolution of strain mapping basically depends on diameter of the probe beam, while can be further downsized by combination with a designed surface label with several tens micrometres. In this study, with the smallest applied strain, the surface strain of the films exceeded 0.5% because of the size and thickness of the films. By employing larger-area, thinner films, smaller surface strain will be measured.

The main purpose of the present paper was to introduce a simple surface-strain analysis technique based on surface-labelled gratings, which we used to pinpoint the delicate nature of strongly bending soft-matter films. In azobenzene-containing liquid-crystalline elastomers, the macroscopic deformation is dictated by a complicated interplay between the optical and mechanical properties (leading to compression-compression mode bending upon photoirradiation). Surface-labelled gratings provide a facile tool that can be used for a detailed experimental analysis of their mechanical/optomechanical response in two dimensions. In substrates used in flexible/stretchable electronics, PDMS-based external surface labels can be used to experimentally verify that the strains acting on the active components are within safety limits. The method can also be used for spatial strain mapping for samples with complex curvature, or can be applied to non-transparent objects, such as thin metal films, by monitoring the strain-induced changes in diffraction angles in a reflection mode.

## Methods

Poly(methylhydrosiloxane) (PMHS) was used as a flexible polymer backbone, and the compounds V4BZM and V4ABM were attached to the backbone as side chains to act as liquid-crystalline and photoresponsive units, respectively. The compound DV9HQ was employed as a crosslinking agent. All of the compounds were synthesized according to published procedures. The chemical structures and the feed ratios of the compounds are shown in Supplementary Fig. S1 online. Supplementary Figures S2 and S3 online show the preparation of unidirectionally aligned azobenzene-containing liquid-crystalline elastomer films with a thickness of 300 μm. The inner wall of the petri dish was covered with a poly(tetrafluoroethylene) (PTFE) film in order to avoid adhesion between the polymer film and the petri dish. The hydrosilylation reaction was conducted at 80°C for 3 h to yield a free-standing, incompletely crosslinked film. Then, a unidirectionally aligned film was fabricated by elongation of the film at room temperature for 24 h, after which the film was heated at 100°C for 24 h to complete the crosslinking reaction.

A transmitted probe beam was normally incident to the screen with a distance *l* = 659 mm. The change in *l* by the film bending was corrected. Applied strain to bend the film was precisely controlled with a stepping motor. The distance between the transmitted and first-diffracted beams, *D*, on the screen was observed with a CCD camera, and with their beam shapes peak-to-peak distance was automatically evaluated with a computer.

## Author Contributions

A.S. designed and directed the project. N.A. and K.S. synthesized the materials. N.A., W.T., S.F. and J.T. prepared the films. N.A. and W.T. performed experiments. A.P., N.A. and A.S. wrote the manuscript, and J.M., M.K. and T.I. had input.

## Supplementary Material

Supplementary Information Supplementary Information
