# Supplementary material for: Facile strain analysis of largely bending films by a surface-labelled grating method
Source: Sci Rep. 2014 Jun 20;4:5377. doi: 10.1038/srep05377 (PMC4064356; doi:10.1038/srep05377)
Supplement: Supplementary Information [file srep05377-s1.pdf]

SUPPLEMENTRY INFORMATION

Facile strain analysis of largely bending films by a surface-labelled grating method

Norihisa Akamatsu[1], Wataru Tashiro[1], Keisuke Saito[1], Jun-ichi Mamiya[1], Motoi Kinoshita[2], Tomiki Ikeda[1,3], Jun Takeya[4], Shigenori Fujikawa[5], Arri Priimagi[1,6] and Atsushi Shishido[1]*

[1] Chemical Resources Laboratory, Tokyo Institute of Technology, R1-12, 4259 Nagatsuta, Midori-ku, Yokohama 226-8503, Japan

[2] Present address: Saitama Institute of Technology, 1690 Fusaiji, Fukaya, Saitama 369-0293, Japan

[3] Present address: Chuo University, 1-13-27 Kasuga, Bunkyo-ku, Tokyo 112-8551, Japan

[4] GSFS, The University of Tokyo, 5-1-5 Kashiwanoha, Kashiwa-shi, Chiba 277-8561, Japan

[5] International Institute for Carbon-Neutral Energy Research (WPI-I2CNER), Kyushu University, 744 Motooka, Nishi-ku, Fukuoka 819-0395, Japan

[6] Department of Applied Physics, Aalto University, P.O. Box 13500, FI-00076 Aalto, Finland

E-mail: ashishid@res.titech.ac.jp

Preparation of azobenzene-containing liquid-crystalline elastomer films.

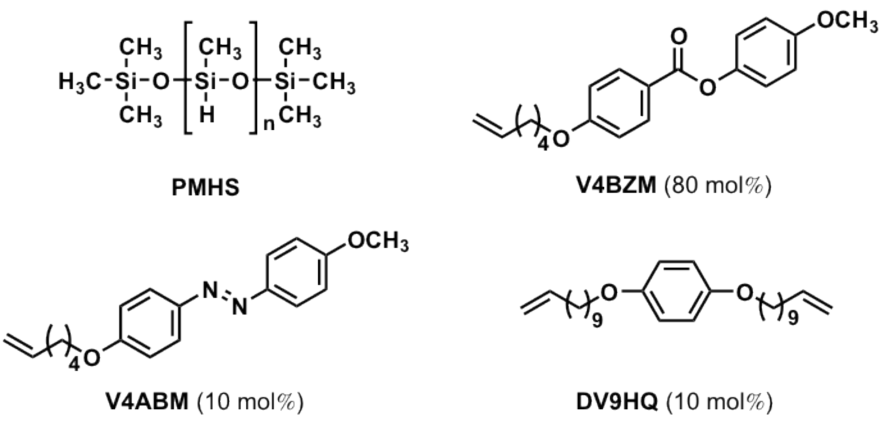

Figure S1. Chemical structures of compounds used in this study.

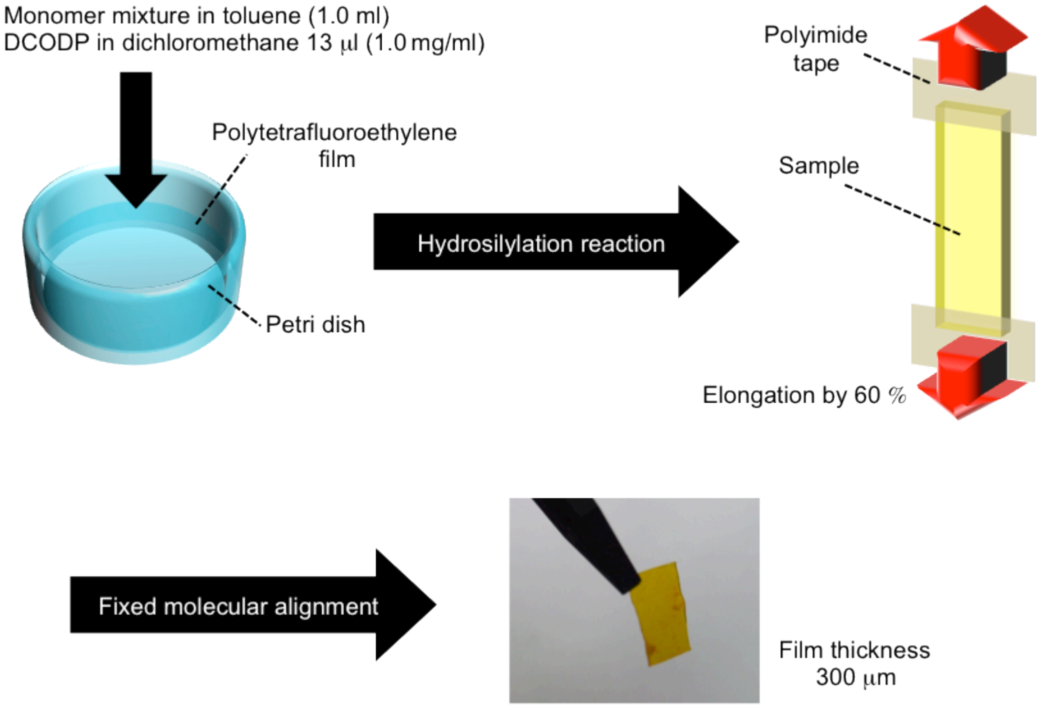

Figure S2. Preparation of azobenzene-containing liquid-crystalline elastomer films by a two-step method.

Evaluation of the molecular alignment.

Figure S3a shows the polarized optical micrographs of azobenzene-containing liquid-crystalline elastomer films. Bright and dark images were subsequently observed when the film was imaged between crossed polarizers and rotated by 45º. The homogeneous molecular alignment was further confirmed by polarized UV-Vis absorption spectroscopy (**Fig. S3b**), from which we deduced that the side chains are preferentially aligned along the elongation direction, with an order parameter of 0.51.

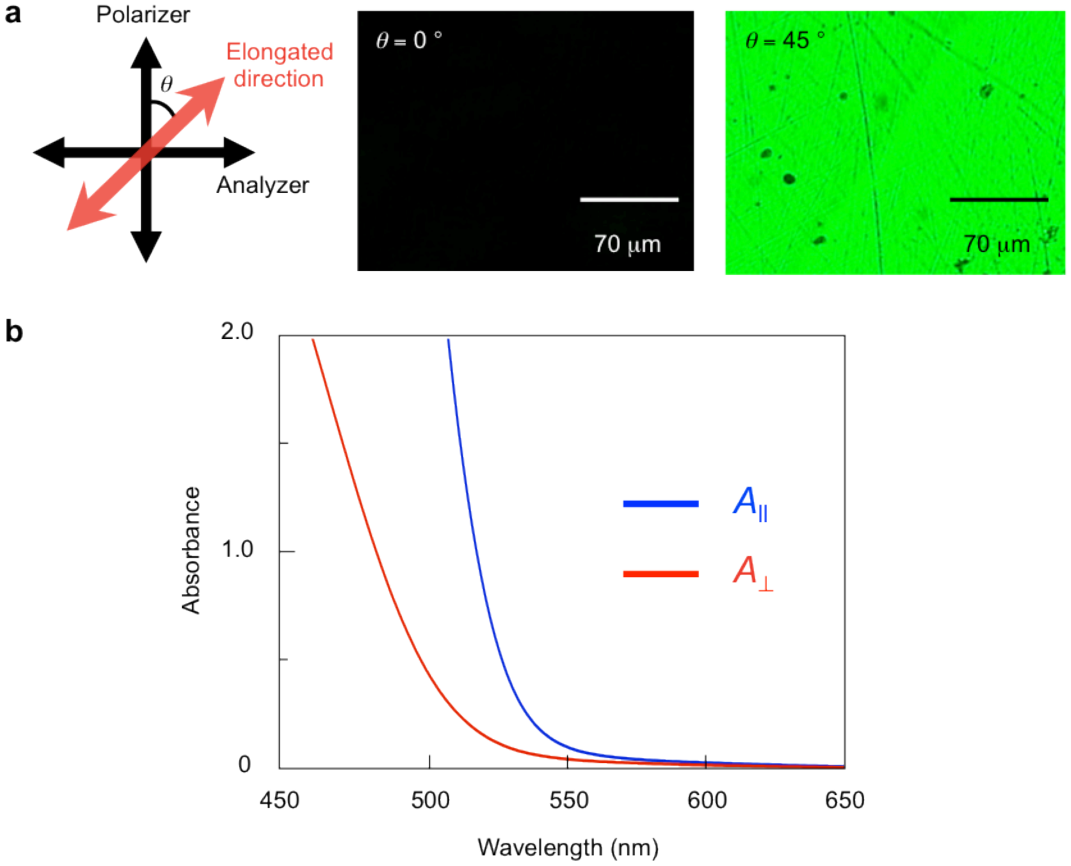

Figure S3. Evaluation of the molecular alignment in the film. a, Polarized optical micrographs of azobenzene-containing liquid-crystalline elastomer films after elongation. **b,** Polarized absorption spectra after the elongation. $A_{\parallel}$ and $A_{\perp}$ denote the absorption parallel and perpendicular to the elongated direction, respectively. The film thickness was 300 μm.

Recording of gratings on surface of the films.

We recorded gratings by trans-cis photoisomerization of azobenzene and the resultant order-disorder change in the molecular alignment upon UV irradiation. The unidirectionally-aligned azobenzene-containing liquid-crystalline elastomer films were labelled with a grating using a photomask with 4 μm grating period. The grating vector of the photomask was set parallel to the molecular alignment direction of the films, and a periodic structure was recorded only in the surface region of the films by irradiation with UV light through a photomask for 5 min. The depth of the periodic structure was confirmed by polarized optical micrograph to be 25 μm (**Fig. S4**). The radiation wavelength and intensity were 366 nm and 10 mW/cm^2, respectively.

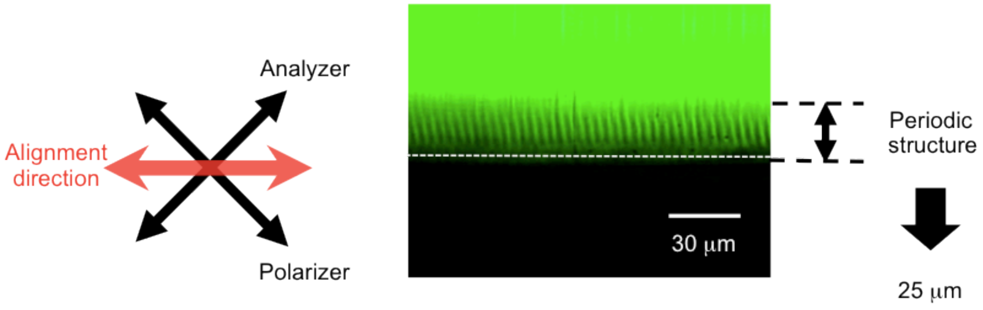

Figure S4. Polarized optical micrograph of the cross-section of the film after the grating formation.

a

Probe spot	Inner surface (%)		Outer surface (%)	
	x	y	x	y
4	-4.2	+0.8	+2.7	-
5	-7.2	+1.2	+3.8	-
6	-4.5	+1.0	+2.3	-

b

Probe spot	Inner surface (%)		Outer surface (%)	
	x	y	x	y
4	-5.4	+2.2	-3.2	+0.9
5	-7.2	+2.9	-3.2	+1.1
6	-5.6	+2.1	-2.9	+0.9

Figure S5. Surface strains of the three spots of a, mechanically-bent and b, photochemically-bent films.

Preparation of PDMS single layer and bilayer films.

Figure S6 shows a method for manufacturing a bilayer film. For single-layer films, the glass cell was set with 500 μm thick spacers between the glass substrate and a silicon substrate. The surface relief grating had 8 μm periodicity which was prepared by photolithography. A mixture of Sylpot and catalyst (10:1 (w/w) for soft film and 5:1 (w/w) for hard film) was inserted into the cell by capillary force to bake for 2 h at 75 ºC. A free-standing PDMS film was obtained by removing the film from the substrates. For bilayer films, a hard PDMS film with a 100 μm thickness was first prepared by the above-mentioned method. After removal of the glass substrate, another glass substrate was set with the 500 μm thick spacers, and a soft PDMS film was prepared on the hard PDMS film to result in a bilayer PDMS film with a total thickness of 500 μm.

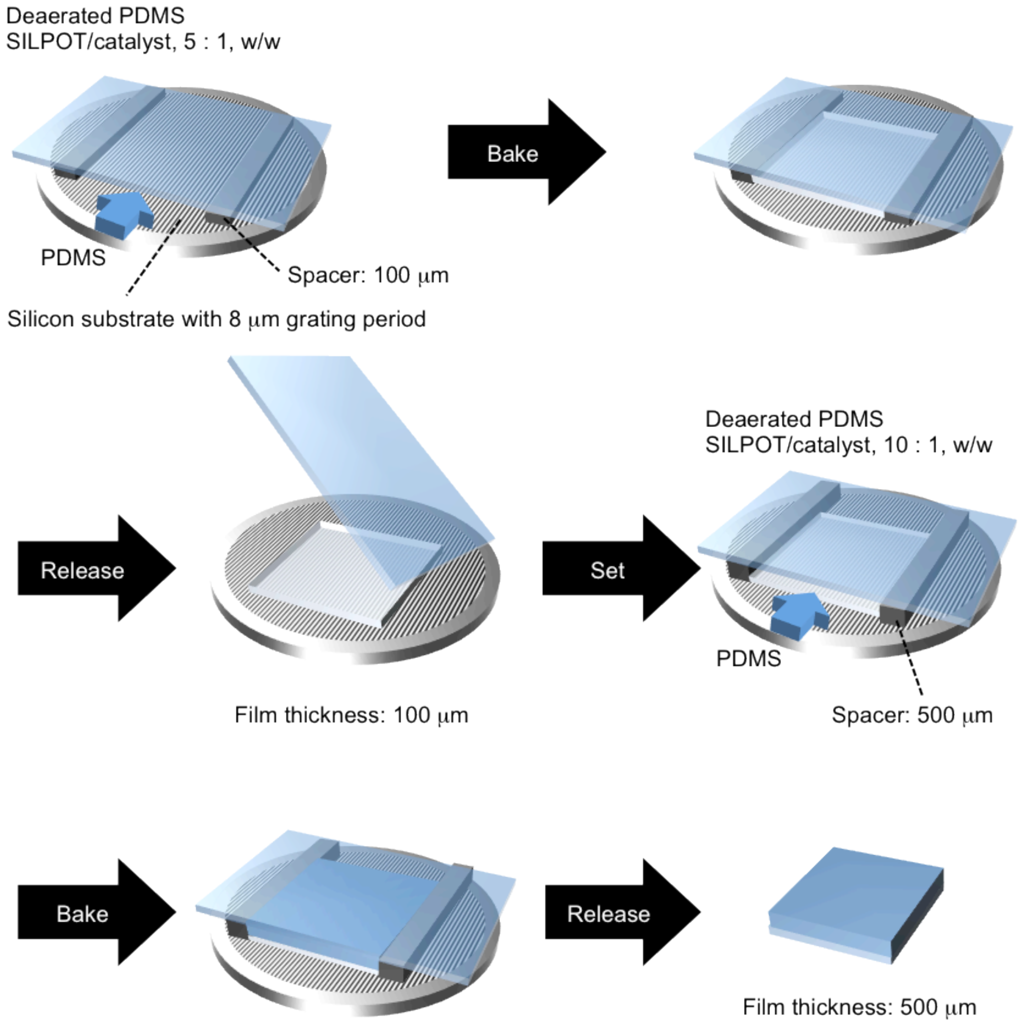

Figure S6. Preparation of bilayer PDMS film.

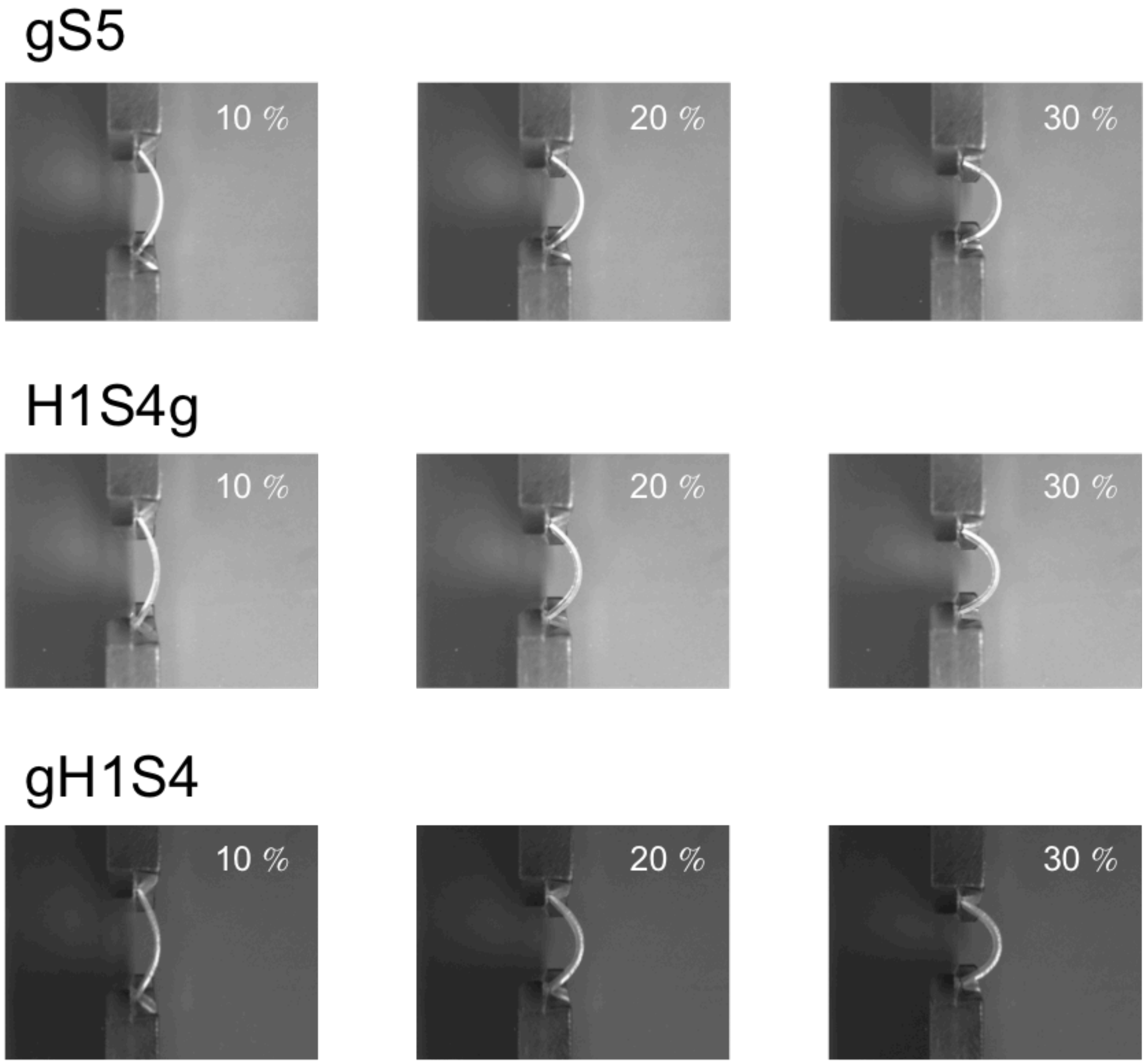

Figure S7. Top-view of mechanically bent single-layer and bilayer PDMS films.

Surface strain analysis of poly(ethylene naphthalate) (PEN) film by means of the PDMS grating label.

Figure S8 shows surface strain measurements of PEN film by means of the soft single-layer PDMS grating label with a thickness of 100 μm. The PDMS label is put on the surface of the PEN film so that the grating is attached with the PEN surface; this allows surface-specific strain evaluation (**Fig. S8a**). With bending of the film by a mechanical stress (**Fig. S8b**), the first-order diffraction angle of the probe beam shifted depending on the applied strain (**Fig. S8a**, **c**). The PDMS

labelling enables the surface strain analysis of various bending films.

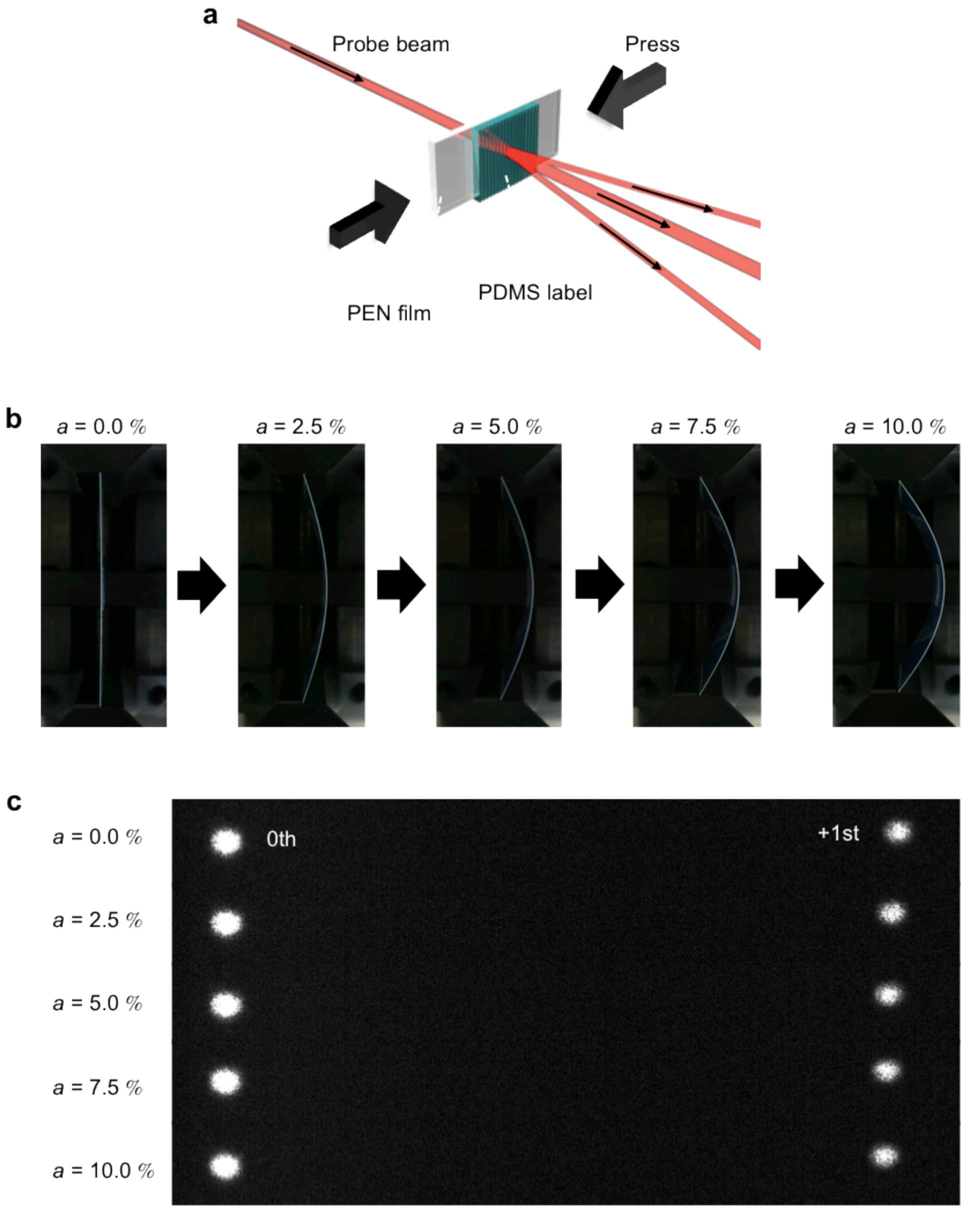

Figure S8. Surface-strain analysis of PEN film by the surface-labelled gratings.

1. Hogan, P. M., Tajbakhsh, A. R., Terentjev, E. M. uv manipulation of order and macroscopic shape in nematic elastomers. *Phys. Rev. E* **65**, 041720 (2002).
2. Küpfer, J., Finkelmann, H. *Makromol. Nematic liquid single crystal elastomers. Chem. Rapid Commun*. **12**, 717 - 726 (1991).
